# Metallic tin quantum sheets confined in graphene toward high-efficiency carbon dioxide electroreduction

**DOI:** 10.1038/ncomms12697

**Published:** 2016-09-02

**Authors:** Fengcai Lei, Wei Liu, Yongfu Sun, Jiaqi Xu, Katong Liu, Liang Liang, Tao Yao, Bicai Pan, Shiqiang Wei, Yi Xie

**Affiliations:** 1 Hefei National Laboratory for Physical Sciences at Microscale, Collaborative Innovation Center of Chemistry for Energy Materials, University of Science & Technology of China, Hefei, Anhui 230026, China; 2 National Synchrotron Radiation Laboratory, University of Science and Technology of China Hefei, Anhui 230029, China; 3 Hefei Science Center of CAS, Hefei, Anhui 230061, China

## Abstract

Ultrathin metal layers can be highly active carbon dioxide electroreduction catalysts, but may also be prone to oxidation. Here we construct a model of graphene confined ultrathin layers of highly reactive metals, taking the synthetic highly reactive tin quantum sheets confined in graphene as an example. The higher electrochemical active area ensures 9 times larger carbon dioxide adsorption capacity relative to bulk tin, while the highly-conductive graphene favours rate-determining electron transfer from carbon dioxide to its radical anion. The lowered tin–tin coordination numbers, revealed by X-ray absorption fine structure spectroscopy, enable tin quantum sheets confined in graphene to efficiently stabilize the carbon dioxide radical anion, verified by 0.13 volts lowered potential of hydroxyl ion adsorption compared with bulk tin. Hence, the tin quantum sheets confined in graphene show enhanced electrocatalytic activity and stability. This work may provide a promising lead for designing efficient and robust catalysts for electrolytic fuel synthesis.

## Abstract (web summary)

Ultrathin metal layers can be highly active carbon dioxide electroreduction catalysts but may also be prone to oxidation. Here, the authors report the fabrication of reactive tin quantum nanosheets confined in graphene and demonstrate their enhanced electrocatalytic activity and stability.

## Graphical abstract

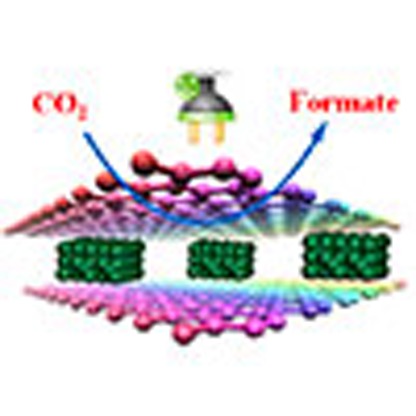

The excessive utilization of fossil fuels provoked the increasing energy crisis and the worsening global climate, which triggered tremendous attention at CO_2_ capture, storage and utilization^1^,^2^,^3^. To address these issues, electrocatalytic reduction of CO_2_ into hydrocarbon fuels is considered as a promising strategy^1^,^2^,^3^, among which metal electrodes, benefiting from high electronic conductivity, show considerable catalytic activities toward CO_2_ electroreduction^4^,^5^,^6^. Nevertheless, the practical applications of these metal electrodes are greatly impeded by the very low energetic efficiencies, which could be primarily ascribed to their extremely low amount of catalytically active sites^7^. Very recently, our study demonstrates that the ultrathin layers of metallic Co exhibit roughly 26 times higher CO_2_ reduction activity relative to bulk Co (ref. 2), profiting from the former's ultralarge fraction of metal atoms exposed on the surface. In spite of such a significant advantage, not all metals could be easily fabricated into ultrathin layers for substantially promoting CO_2_ reduction performances. For some metals with higher chemical reactivity, while exposing ultrahigh amount of surface atoms, they tend to be very unstable at ambient conditions and will be oxidized in a non-controlled manner^1^,^5^,^6^,^8^, which would lead to the loss of electronic conductivity and consequently cause a rapid decay in the cyclability. As such, developing a new strategy for stabilizing the ultrathin layers of highly reactive metals is highly imperative for both fundamental research and practical application in CO_2_ electroreduction.

Motivated by the above consideration, we construct a material model of ultrathin metal layers confined in graphene, in which the protection of graphene could avoid the oxidation of the highly reactive metals at ambient atmosphere (Fig. 1). The metal ultrathin layers could not only serve as a ‘spacer' to favour electrolyte diffusion into the matrix of graphene^9^, but also afford abundant surface atoms to act as the active sites for efficient CO_2_ adsorption^10^,^11^, hence providing the prerequisite to involve the following reduction reactions. That is to say, in our designed sandwich-like structure, there is enough space between the graphene layers and hence the electrolyte can freely diffuse into/out the deep matrix of graphene. In this case, CO_2_ could diffuse into the graphene layers through dissolving in the electrolyte and simultaneously the products could also be transported out of the graphene layers by virtue of the freely flowing electrolyte. Moreover, the highly-conductive graphene endures fast electron penetration to the metal ultrathin layers^12^, which hence favours the rate-determining electron transfer from CO_2_ to CO_2_^−^ intermediate. In addition, as revealed by X-ray absorption fine structure spectroscopy (XAFS) in our previously systematic studies^13^,^14^, the exposed surface metal atoms would possess lowered coordination number compared with interior atoms, in which the low-coordinated metal atoms with many dangling bonds could help to stabilize the CO_2_^−^ intermediate, hence lowering the overall activation energy barrier and remarkably improving the catalytic activity. Furthermore, the intimate contact between ultrathin metal layers and graphene in a sandwich-like fashion could ensure excellent long-term stability.

## Results

### Characterizations of Sn quantum sheets confined in graphene

**Figure 2:**
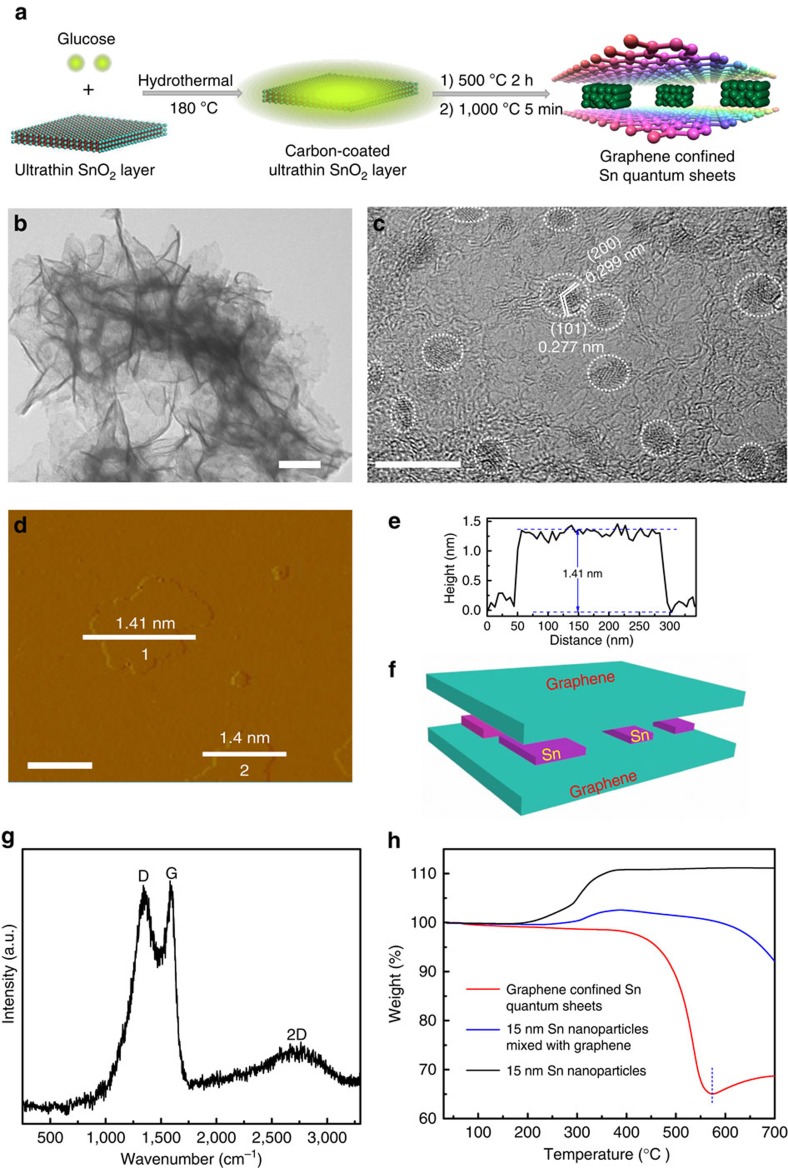
Formation process and characterizations of the Sn quantum sheets confined in graphene. (**a**) Scheme illustration for the formation of Sn quantum sheets confined in graphene, (**b**) TEM image, (**c**) HRTEM image, (**d**–**f**) AFM image, the corresponding height profile and scheme illustration and (**g**) micro-Raman spectrum of the Sn quantum sheets confined in graphene. (**h**) TG analysis of Sn quantum sheets confined in graphene, 15 nm Sn nanoparticles and 15 nm Sn nanoparticles mixed with graphene. The scale bars in (**b**–**d**) are 100, 10 and 200 nm, respectively. The inset circles in (**c**) denote the presence of Sn quantum sheets.

Of note, the low cost and environmentally benign Sn is not only one of the most active metals, but also possesses selective reduction of CO_2_ to formate^5^,^8^,^15^, which enables it to be a highly reactive metal candidate for investigating the corresponding CO_2_ reduction performances. Therefore, it is highly warranted for controllably synthesizing Sn ultrathin layers in the confined space of graphene. Herein, metallic Sn quantum sheets confined in graphene were first successfully synthesized through a spatially confined reduction strategy (Fig. 2a). The as-prepared ultrathin SnO_2_ layers^16^ were initially coated with amorphous carbon through a hydrothermal method. Hence, after subsequent temperature-programmed calcination for the carbon-coated ultrathin SnO_2_ layers, the amorphous carbon turned into graphene, while the ultrathin SnO_2_ layers simultaneously reduced into monodispersed Sn quantum sheets in the confined space of graphene, benefiting from the short calcination time and rapid subsequent cooling in an inert atmosphere of Ar gas.

With regard to the precise characterization of ultrathin Sn layers, it encounters many great difficulties since the lack of long-range order in the third dimensionality causes the typical X-ray diffraction technique unable to identify the spatial atomic distribution, while the XPS spectrum is only a surface-sensitive spectroscopic technique^7^. In this regard, high-resolution transmission electron microscopy (HRTEM) image and synchrotron radiation XAFS spectroscopy were utilized to characterize the phase and atomic structure of the synthetic Sn quantum sheets. Transmission electron microscopy (TEM) image in Fig. 2b shows the extremely thin sheet-like morphology of graphene, in which the Raman bands at around 1,352, 1,593 and 2,700 cm^−1^ demonstrated the formation of graphene (Fig. 2g)^17^. In addition, HRTEM image in Fig. 2c also shows that abundant two-dimensional (2D) sheets nearly monodispersed on the graphene, while the interplanar spacing of 0.299 and 0.277 nm as well as the corresponding dihedral angle of 61.4° could index them to be tetragonal Sn. It should be noted that the Sn quantum sheets were encapsulated in graphene rather than exposed on the outer surface (Fig. 2f), which could be further implied by the thermogravimetric (TG) result in Fig. 2h. The TG analysis in air showed a rapid weight loss between 390 and 570 °C, corresponding to the decomposition of gaphene, and a weight increase at above 570 °C, owing to the oxidation of metallic Sn, which indicated that the protection of graphene enabled the Sn quantum sheets to be stable in air up to 570 °C. Contrastingly, both the synthetic 15 nm Sn nanoparticles and their mixture with graphene showed a weight increase at above ca. 200 °C (see Methods for details), indicating that the metallic Sn is easily oxidized without the protection of graphene interlayer. As a result, the presence of graphene interlayer not only avoided the oxidation of metallic Sn, but also prevented the aggregation of quantum sized Sn sheets. Moreover, as revealed by the atomic force microscopic image and the corresponding height profiles in Fig. 2d–e, the sandwich-like structure possessed an average thickness of ca. 1.4 nm, which indicated that the thickness of either Sn or graphene layer was much less than 1.4 nm. Accordingly, the above results demonstrated the formation of monodisperse Sn quantum sheets confined in graphene.

**Figure 3:**
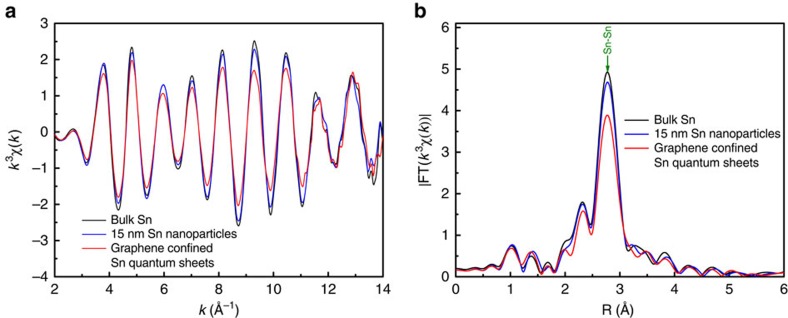
Synchrotron radiation XAFS measurements. (**a**) Sn K-edge extended XAFS oscillation function *k*^3^χ(*k*), (**b**) the corresponding Fourier transforms FT(*k*^3^χ(*k*)) for the graphene confined Sn quantum sheets, 15 nm Sn nanoparticles and bulk Sn, respectively.

To further identify the phase and unravel the local atomic arrangements of the synthetic Sn quantum sheets, synchrotron radiation XAFS measurements were performed. As shown in Fig. 3a,b, the Sn quantum sheets confined in graphene exhibited a strong FT peak of Sn–Sn metallic bonding at ca. 2.77 Å, while they did not possess the peaks for Sn^4+^–O at ca. 1.45 Å and Sn–O–Sn at above 3 Å, which undoubtedly confirmed that the Sn quantum sheets were well protected from oxidation through spatially confining in the graphene. In addition, their Sn K-edges *k*^3^χ(*k*) oscillation curves in Fig. 3a show obvious differences relative to the 15 nm Sn nanoparticles and bulk counterpart (Supplementary Figs 1–3), which suggested their distinct local atomic arrangement further verified by their R-space curves in Fig. 3b. According to the high-quality extended XAFS spectra, least-squares fittings were further conducted and the obtained quantitative results are shown in Table 1. For the Sn quantum sheets confined in graphene, the coordination numbers of Sn–Sn reduced from 2 and 4 to 1.4 and 2.7, while their disorder degrees increased significantly as compared with the 15 nm Sn nanoparticles and bulk counterpart. This suggested that the reduced size resulted in many dangling bonds as well as a noticeable distortion on the surface of Sn quantum sheets, which would contribute to their high catalytic activity along with excellent stability^7^,^13^,^14^. Altogether, both the HRTEM and XAFS results disclosed that the synthetic Sn quantum sheets were free of oxidation under the effective umbrella of graphene.

### Electroreduction of CO_2_ into hydrocarbon fuels

**Figure 4:**
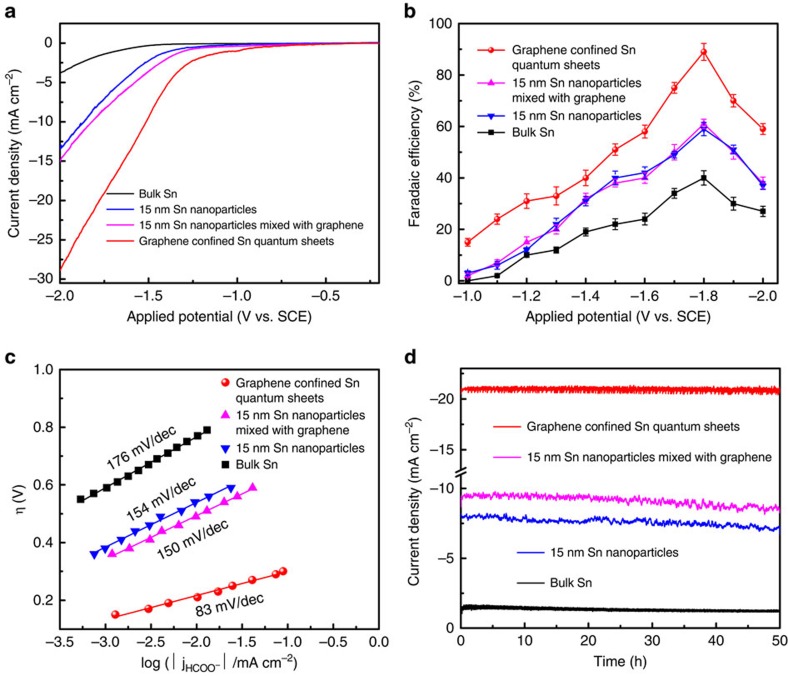
CO_2_ electroreduction performances. (**a**) Linear sweep voltammetric curves in the CO_2_-saturated 0.1 M NaHCO_3_ aqueous solution, (**b**) Faradaic efficiencies for formate at each applied potentials for 4 h, (**c**) Tafel plots for producing formate, (**d**) Chrono-Amperometry results at the potentials of −1.8 V versus SCE for the Sn quantum sheets confined in graphene, 15 nm Sn nanoparticles mixed with graphene, 15 nm Sn nanoparticles and bulk Sn. The error bars in **b** represent the standard deviations of five independent measurements of the same sample.

Benefiting from the lowered coordination number, obvious structure distortion as well as surface conductive graphene, the Sn quantum sheets confined in graphene were expected to give substantially promoted CO_2_ electroreduction performances in comparison with the Sn nanoparticles as well as the bulk counterpart. To verify the higher activity of the unique sandwich-like 2D structure, CO_2_ reduction activities for the Sn quantum sheets confined in graphene, 15 nm Sn nanoparticles mixed with graphene, 15 nm Sn nanoparticles and bulk Sn were determined in CO_2_-saturated 0.1 M NaHCO_3_ solution. As revealed by linear sweep voltammetry (LSV) in Fig. 4a, the Sn quantum sheets confined in graphene exhibited the highest catalytic activity among the above four samples. For instance, the Sn quantum sheets confined in graphene displayed a current density of 21.1 mA cm^−2^ at −1.8 V versus SCE, which was roughly 2, 2.5 and 13 times larger than that of the 15 nm Sn nanoparticles mixed with graphene, 15 nm Sn nanoparticles and bulk Sn, respectively. In addition, the Sn quantum sheets confined in graphene exhibited an onset potential of −0.85 V versus SCE, which was much positive relative to other three samples (Fig. 4a). This strongly demonstrated the simple addition of graphene could only slightly mend the catalytic activity, while the formation of unique sandwich construction can help to remarkably promote the activity.

To analyse the electroreduction products, stepped-potential electrolyses at each given potential for 4 h were performed to periodically quantify the liquid products by ^1^H nuclear magnetic resonance spectroscopy and the gas products by an Agilent Technologies 7890B gas chromatograph^2^. As revealed by the faradaic efficiency for formate production in Fig. 4b, one can clearly observe that the Sn quantum sheets confined in graphene behaved obviously higher faradaic efficiency relative to other three samples in the whole applied potentials. At the potential of −1.8 V versus SCE, the Sn quantum sheets confined in graphene attained a maximum faradaic efficiency of 89%, which was roughly 1.45, 1.5 and 2 times higher than that of the 15 nm Sn nanoparticles mixed with graphene, 15 nm Sn nanoparticles and bulk Sn, denoting the superior activity of their unique structure. In addition, the faradaic efficiency for gas products in Supplementary Fig. 4 reveal that the main gas products are the H_2_, while CO accounted for the remaining products. Of note, the above four samples achieved the maximum formate faradaic efficiency at the similar potentials, which could be ascribed to the mass transport limitation of CO_2_ as well as the competitive H_2_ evolution reaction^8^,^18^. To obtain further insights into these electrocatalysts, their Tafel plots were investigated and presented in Fig. 4c. The resulting Tafel slope of the Sn quantum sheets confined in graphene was 83 mV dec^−1^, much smaller than that of the 15 nm Sn nanoparticles mixed with graphene, 15 nm Sn nanoparticles and bulk Sn, respectively. The smaller Tafel slope of the Sn quantum sheets confined in graphene is very advantageous for practical applications, since it will lead to a much faster increment of CO_2_ reduction rate with increasing overpotential^10^. Besides the activity of CO_2_ electroreduction, electrochemical stability is another significant criterion to evaluate an advanced electrocatalyst. Hence, continuous CO_2_ reduction was performed at −1.8 V versus SCE for 50 h in order to probe the durability of the above electrocatalysts. The Sn quantum sheets confined in graphene did not show any obvious decay in the current densities, while their Faradaic efficiency for producing formate was always larger than 85% during the long test period of 50 h, indicative of their very favourable stability (Fig. 4d). Contrastingly, all the 15 nm Sn nanoparticles mixed with graphene, 15 nm Sn nanoparticles and bulk Sn exhibited relatively poor long-term stability. Therefore, the Sn quantum sheets confined in graphene have sufficient potential as a promising catalyst for electrocatalytic CO_2_ conversion with a superior formate production rate as well as a prolonged stability.

## Discussion

**Figure 5:**
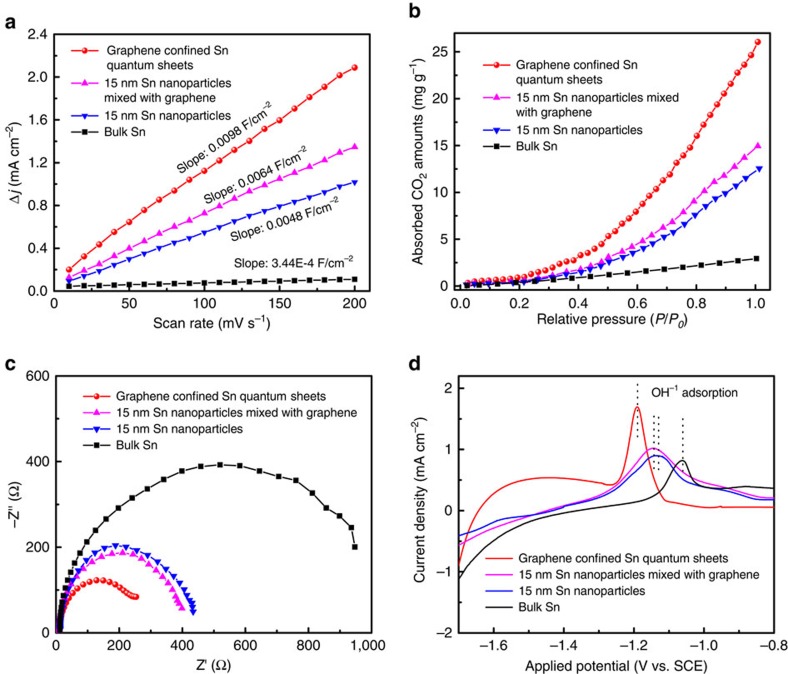
Advantages of the Sn quantum sheets confined in graphene. (**a**) Charging current density differences plotted against scan rates; (**b**) CO_2_ adsorption isotherms; (**c**) Nyquist plots; (**d**) single oxidative LSV scans in N_2_-saturated 0.1 M NaOH for the Sn quantum sheets confined in graphene, 15 nm Sn nanoparticles mixed with graphene, 15 nm Sn nanoparticles and bulk Sn.

With regard to the notable enhancement of catalytic activity for the Sn quantum sheets confined in graphene, the increase in electrochemical active surface area (ECSA) may be one of the main contributors since larger ECSA could afford more catalytically active sites^2^. According to the measured double-layer capacitance in Fig. 5a, the ECSA for the Sn quantum sheets confined in graphene was estimated to be roughly 1.5, 2 and 28 times higher than that of the 15 nm Sn nanoparticles mixed with graphene, 15 nm Sn nanoparticles and bulk Sn. The increased ECSA for the Sn quantum sheets confined in graphene suggested their remarkably larger amount of active sites, which endowed them with increased CO_2_ adsorption capacity, verified by the corresponding CO_2_ adsorption isotherms in Fig. 5b. For instance, the amount of CO_2_ adsorption capacity for the Sn quantum sheets confined in graphene could reach 26.1 mg g^−1^ at 1 atm, which is roughly 1.75, 2 and 9 times higher than that of the 15 nm Sn nanoparticles mixed with graphene, 15 nm Sn nanoparticles and bulk counterpart. Compared with the 15 nm Sn nanoparticles, the 15 nm Sn nanoparticles mixed with graphene possessed a slightly increased CO_2_ adsorption amount, which suggested that the graphene played a limited role in increasing the CO_2_ adsorption. Thus, the 28 times higher ECSA for the Sn quantum sheets confined in graphene, largely coming from the high surface area graphene, only led to ca. 9 times higher CO_2_ adsorption capacity relative to bulk Sn, and hence only resulted in 13 times higher current density. Moreover, the lowered Sn–Sn coordination number for the Sn quantum sheets confined in graphene, revealed by XAFS fitting results in Fig. 3, further indicated their higher intrinsic catalytic activity, which could be further demonstrated by their Tafel slope lowered from 176 to 83 mV dec^−1^ relative to bulk Sn in Fig. 4c. Moreover, the presence of highly-conductive graphene could enhance the overall electronic conductivity, which was confirmed by their lowest interfacial charge-transfer resistance in Fig. 5c, hence ensuring fast electron transfer to CO_2_ for forming CO_2_^−^ radiacal anion intermediate. Of note, the first electron transfer step of CO_2_ + e → CO_2_^−^ is generally regarded as the rate-determining step^1^,^2^,^4^,^5^,^6^, in which stabilization of the CO_2_^−^ intermediate plays a fundamental role in CO_2_ reduction into formate. To testify the binding affinity of CO_2_^−^ on the four samples, adsorption of OH^−^ as a surrogate for CO_2_^−^ was conducted by oxidative LSV scans under a N_2_-bubbled 0.1 M NaOH electrolyte. The results in Fig. 5d revealed that the potential for surface OH^−^ adsorption on the Sn quantum sheets confined in graphene was 0.05, 0.06 and 0.13 V lower than that of the 15 nm Sn nanoparticles mixed with graphene, 15 nm Sn nanoparticles and bulk Sn. This illustrated the Sn quantum sheets confined in graphene possessed stronger adsorption affinity of OH^−^, and hence they could efficiently stabilize the CO_2_^−^ intermediate^8^,^19^, finally facilitating formate production. Therefore, the above results strongly demonstrated that the simple addition of graphene could only slight improve the CO_2_ reduction performances, while the graphene interlayer confined Sn quantum sheets could fully optimize the crucial processes during CO_2_ electroreduction into formate.

In conclusion, we first put forward an ideal material model of ultrathin metal layers confined in graphene, in which the protection of graphene could avoid the oxidation of the highly reactive metals in air. As an example, highly reactive metallic Sn quantum sheets, confined in few-layered graphene, are first successfully synthesized through a spatially confined reduction strategy. Synchrotron radiation XAFS results disclose that Sn–Sn coordination numbers for the Sn quantum sheets confined in graphene reduce from 2 and 4 to 1.4 and 2.7 compared with that of bulk counterpart, which implies the former's higher intrinsic catalytic activity, further demonstrated by their lowered Tafel slope from 176 to 83 mV dec^−1^. The remarkably increased ECSA for the Sn quantum sheets confined in graphene could afford larger amount of active sites to efficiently adsorb CO_2_, further confirmed by their 1.75, 2 and 9 times higher CO_2_ adsorption capacity compared with the 15 nm Sn nanoparticles mixed with graphene, 15 nm Sn nanoparticles and bulk Sn. The lowest interfacial charge-transfer resistance, benefiting from the highly-conductive graphene, facilitates the rate-limiting electron transfer from CO_2_ to CO_2_^−^ intermediate. In addition, compared to the 15 nm Sn nanoparticles mixed with graphene, 15 nm Sn nanoparticles and bulk Sn, the 0.05, 0.06 and 0.13 V lowered potential of OH^−^ adsorption indicate that the Sn quantum sheets confined in graphene possess the strongest adsorption affinity of CO_2_^−^ intermediate, and hence they efficiently stabilize the CO_2_^−^ intermediate, thus definitely lowering the overall reaction barrier. As an outcome, the Sn quantum sheets confined in graphene display a current density of 21.1 mA cm^−2^ at −1.8 V versus SCE, which is roughly 2, 2.5 and 13 times larger than that of the 15 nm Sn nanoparticles mixed with graphene, 15 nm Sn nanoparticles and bulk Sn. Also, the formate Faradaic efficiency for Sn quantum sheets confined in graphene is always larger than 85% during the long test period of 50 h. Thus, the present work demonstrates that the unique structure of highly reactive metal quantum sheets confined in graphene could fully optimize CO_2_ electroreduction performances.

## Methods

### Synthesis of Sn quantum sheets confined in grapheme

In a typical synthesis, 10 mg ultrathin SnO_2_ layers, synthesized according to our previous work (Angew. Chem. Int. Ed. 2013, 52, 10,569), were dispersed in 40 ml 0.03 M aqueous glucose solution under mild stirring for 30 min. Then the above mixture was transferred into a 50 ml Teflon-lined autoclave, sealed and heated at 180 °C for 10 h. After the initial hydrothermal method, the ultrathin SnO_2_ layers were homogenerously encapsulated in the carbon layer. Then, the carbon-coated ultrathin SnO_2_ layers were initially annealed at 500 °C for 2 h, and then immediately annealed at 1,000 °C for 5 min, and hence cooled down rapidly (within 1 min) to room temperature under the protection of argon atmosphere. During this temperature-programed process, the amorphous carbon turned into graphene, while the ultrathin SnO_2_ layers simultaneously reduced into monodispersed Sn quantum sheets in the confined space of graphene, thus forming a sandwich-like structure. By contrast, for the synthesis of 15 nm Sn nanoparticles mixed with graphene, the 15 nm Sn nanoparticles, synthesized according to the ref. of Nanotechnology, 2011, 22, 22,5701, were physically mixed with the commercial graphene under mild stirring for 1 h. Then, the 15 nm Sn nanoparticles could be dispersed on the surface of graphene.

### Synthesis of bulk Sn

In a typical synthesis, 50 mg ultrathin SnO_2_ layers, synthesized according to ref. Angew. Chem. Int. Ed. 2013, 52, 10,569, were annealed at 500 °C for 5 h in H_2_ atmosphere and then cooled down to room temperature. The obtained powders were collected for further characterization.

### Characterization

TEM images and high-resolution TEM image were performed by using a JEOL-2010 TEM with an acceleration voltage of 200 kV. The Sn *K*-edge XAFS was measured at the Shanghai Synchrotron Radiation Factory and the National Synchrotron Radiation Laboratory, China. Thermal gravimetric analysis (TGA) of the as-synthesized samples were carried out on a Shimadzu TA-50 thermal analyser at a heating rate of 10 °C min^−1^ from room temperature to 700 °C in air. The liquid products were quantified by nuclear magnetic resonance (Bruker AVANCE AV III 400) spectroscopy. CO_2_ adsorption isotherms measurements for all the synthetic samples were carried out by using an automatic microporous physical and chemical gas adsorption analyser (ASAP 2020M+C).

### Electrochemical measurements

Electrochemical measurements were implemented in a three-electrode system at an electrochemical station (CHI660E). The working electrode was glassy carbon electrode. The platinum gauze and the saturated calomel electrode (SCE) reference electrode served as counter and the reference electrodes, respectively. In a typical prepared procedure of the working electrode, 3 μl of the homogeneous ink, which was prepared by dispersing 5 mg sample and 40 μl Nafion solution (5 wt%) in 1 ml water-ethanol solution with volume ratio of 3:1, was loaded onto a glassy carbon electrode with 3 mm diameter. For CO_2_ reduction experiments, LSV with a scan rate of 20 mV s^−1^ was carried out in CO_2_-saturated 0.1 M NaHCO_3_ solution (60 ml) (The NaHCO_3_ electrolyte was purged with CO_2_ for 30 min prior to the measurement.). ECSA was determined by measuring the capacitive current associated with double-layer charging from the scan-rate dependence of CVs. The *C*_dl_ was estimated by plotting the Δ*j* (*j*_a_−*j*_c_) at −0.75 V versus SCE against the scan rate, where the Δ*j* could be acquired by Cyclic voltammetry measurement under the potential windows of −0.8∼−0.7 V versus SCE (0.1 M NaHCO_3_ solution).

### EXAFS experimental details

Sample of 20 mg was homogeneously mixed with 100 mg graphite and hence pressed into circular pellets with a diameter of 10 mm for further extended X-ray absorption fine structure (EXAFS) measurement under ambient conditions. Then, the XAFS measurements were performed at BL14W1 station in Shanghai Synchrotron Radiation Facility, China. The storage rings of SSRF were operated at 3.5 GeV with the maximum current of 210 mA. Si(311) double-crystal monochromator crystals were used to monochromatize the X-ray beam. The energy resolution at Sn K-edge was about 2 eV. XAFS data were collected in transmission in the energy range from −200 below to 1,000 eV above the Sn K-edge. The detuning was done by 30% to remove harmonics.

### Data availability

The authors declare that the data supporting the findings of this study are available within the article and its Supplementary Information files and from the corresponding author upon reasonable request.

## Additional information

**How to cite this article:** Lei, F. *et al.* Metallic tin quantum sheets confined in graphene toward high-efficiency carbon dioxide electroreduction. *Nat. Commun.* 7:12697 doi: 10.1038/ncomms12697 (2016).

## Supplementary Material

Supplementary Information Supplementary Figures 1-4

## Figures and Tables

**Figure 1:**
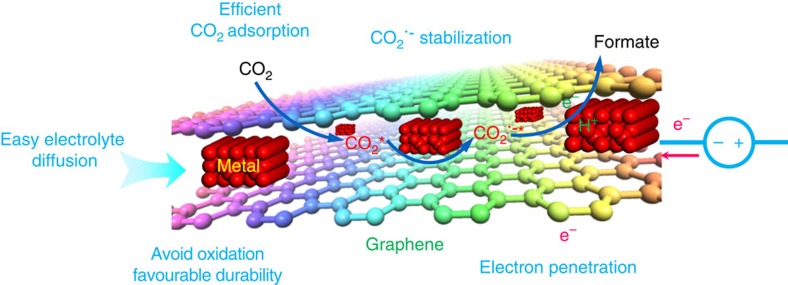
Scheme. Schematic illustration depicts the several advantages of ultrathin metal layers confined in graphene for CO_2_ electroreduction into hydrocarbon fuels.

**Table 1:** EXAFS curve-fitting results.

Sample	Path	*N*	*R* (Å)	σ^2^ (10^−3^Å^2^)	Δ*E*_0_ (eV)
Bulk Sn	Sn–Sn1	4.0	3.02	9.1	3.6
	Sn–Sn2	2.0	3.18	10.5	3.6
15 nm Sn nanoparticles	Sn–Sn1	4.0	3.02	9.7	4.0
	Sn–Sn2	2.0	3.18	12.0	4.0
Graphene confined Sn quantum sheets	Sn–Sn1(surface)	2.7	3.01	10.8	4.3
	Sn–Sn1(interior)	4.0	3.02	9.3	4.3
	Sn–Sn2(surface)	1.4	3.17	14.0	4.3
	Sn–Sn2(interior)	2.0	3.18	10.7	4.3

EXAFS, extended X-ray absorption fine structure.

Structural parameters around Sn atoms extracted from EXAFS curve-fitting for the graphene confined Sn quantum sheets, 15 nm Sn nanoparticles and bulk Sn, respectively.
