# Supplementary material for: Metallic tin quantum sheets confined in graphene toward high-efficiency carbon dioxide electroreduction
Source: Nat Commun. 2016 Sep 2;7:12697. doi: 10.1038/ncomms12697 (PMC5025773; doi:10.1038/ncomms12697)
Supplement: Supplementary Information — Supplementary Figures 1-4 [file ncomms12697-s1.pdf]

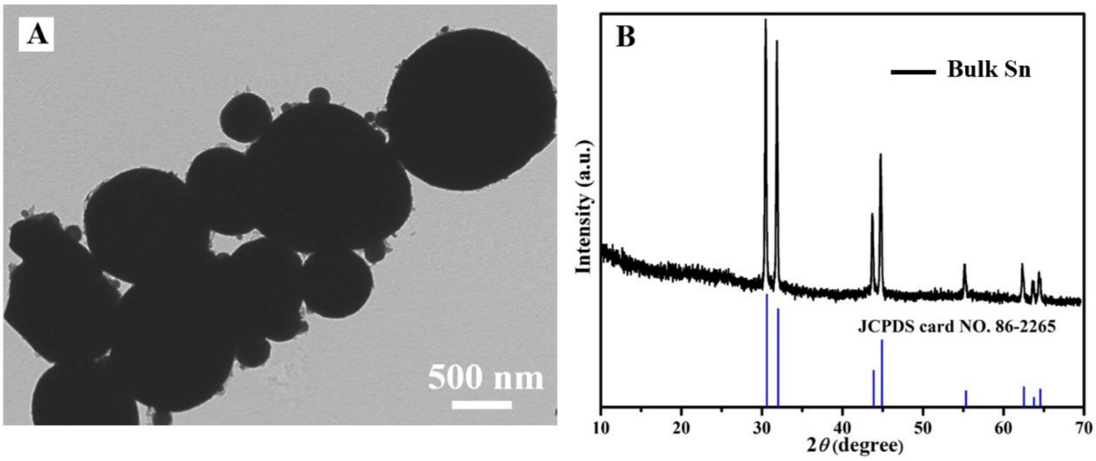

Supplementary Figure 1. Characterizations. TEM image and XRD pattern for bulk Sn.

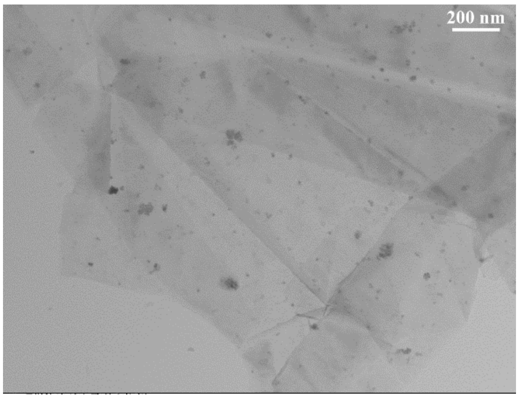

Supplementary Figure 2. Characterizations. TEM image of the 15 nm Sn nanoparticles mixed with graphene.

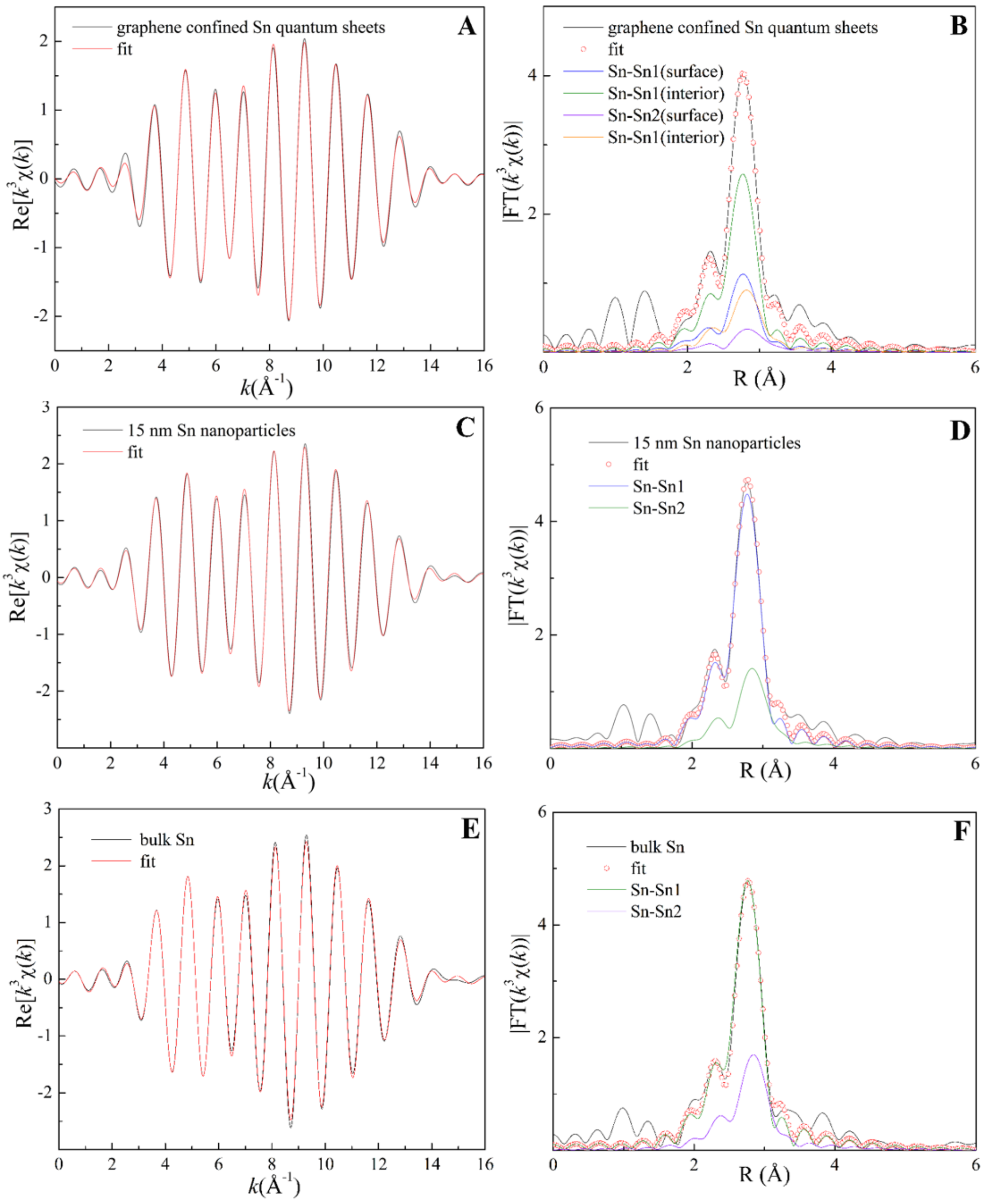

Supplementary Figure 3. EXAFS fitting results. The EXAFS fitting results for the Sn *K*-edge for (A-B) the graphene confined Sn quantum sheets, (C-D) 15 nm Sn nanoparticles and (E-F) bulk Sn, respectively.

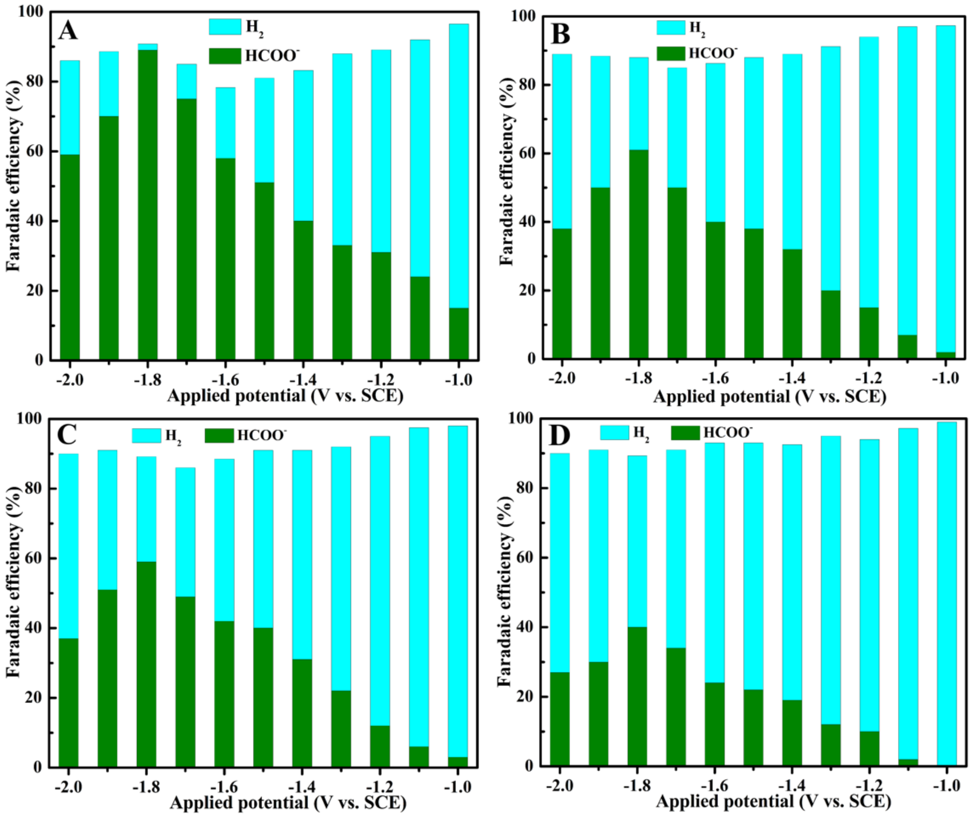

Supplementary Figure 4. Faradaic efficiencies. Faradaic efficiencies for formate and H_2 at each applied potentials for 4 h for (A) the Sn quantum sheets confined in graphene, (B) 15 nm Sn nanoparticles mixed with graphene, (C) 15 nm Sn nanoparticles and (D) bulk Sn, respectively. Of note, CO accounted for the remaining reduction productions in the above four samples.
